# *Anopheles* bionomics, insecticide resistance mechanisms, and malaria transmission in the Korhogo area, northern Côte d’Ivoire: a pre-intervention study

**DOI:** 10.1051/parasite/2019040

**Published:** 2019-07-12

**Authors:** Barnabas Zogo, Dieudonné Diloma Soma, Bertin N’Cho Tchiekoi, Anthony Somé, Ludovic P. Ahoua Alou, Alphonsine A. Koffi, Florence Fournet, Amal Dahounto, Baba Coulibaly, Souleymane Kandé, Roch Kounbobr Dabiré, Lamine Baba-Moussa, Nicolas Moiroux, Cédric Pennetier

**Affiliations:** 1 Institut Pierre Richet (IPR), Institut National de Santé Publique (INSP) BP 1500 Bouaké Côte d’Ivoire; 2 Maladies Infectieuses et Vecteurs : Ecologie, Génétique, Evolution et Contrôle (MIVEGEC), Univ Montpellier, CNRS, IRD BP 64501 Montpellier France; 3 Faculté des Sciences et Techniques (FAST), Université d’Abomey-Calavi BP 1604 Cotonou Benin; 4 Institut de Recherche en Sciences de la Santé (IRSS) BP 545 Bobo Dioulasso Burkina Faso; 5 Institut Supérieur des Sciences de la Santé, Université Nazi Boni BP 1091 Bobo-Dioulasso Burkina Faso

**Keywords:** Malaria, Vectors, Resistance, Intensity of transmission

## Abstract

A better understanding of malaria transmission at a local scale is essential for developing and implementing effective control strategies. In the framework of a randomized controlled trial (RCT), we aimed to provide an updated description of malaria transmission in the Korhogo area, northern Côte d’Ivoire, and to obtain baseline data for the trial. We performed human landing collections (HLCs) in 26 villages in the Korhogo area during the rainy season (September–October 2016, April–May 2017) and the dry season (November–December 2016, February–March 2017). We used PCR techniques to ascertain the species of the *Anopheles gambiae* complex, *Plasmodium falciparum* sporozoite infection, and insecticide resistance mechanisms in a subset of *Anopheles* vectors. *Anopheles gambiae s.l.* was the predominant malaria vector in the Korhogo area. Overall, more vectors were collected outdoors than indoors (*p* < 0.001). Of the 774 *An. gambiae s.l.* tested in the laboratory, 89.65% were *An. gambiae s.s.* and 10.35% were *An. coluzzii*. The frequencies of the *kdr* allele were very high in *An. gambiae s.s.* but the *ace-1* allele was found at moderate frequencies. An unprotected individual living in the Korhogo area received an average of 9.04, 0.63, 0.06 and 0.12 infected bites per night in September–October, November–December, February–March, and April–May, respectively. These results demonstrate that the intensity of malaria transmission is extremely high in the Korhogo area, especially during the rainy season. Malaria control in highly endemic areas such as Korhogo needs to be strengthened with complementary tools in order to reduce the burden of the disease.

## Abstract (fr)

Une meilleure connaissance de la transmission du paludisme à l’échelle locale est essentielle pour élaborer et mettre en œuvre des stratégies de lutte efficaces. Dans le cadre d’un essai contrôlé randomisé, nous avons pour objectifs de fournir une description actualisée de la transmission du paludisme dans la zone de Korhogo, au nord de la Côte d’Ivoire, et de collecter les données de base pour l’essai. Nous avons capturé les moustiques sur des volontaires humains dans 26 villages de la zone de Korhogo pendant la saison pluvieuse (septembre–octobre 2016, avril–mai 2017) et la saison sèche (novembre–décembre 2016, février–mars 2017). À l’aide des techniques de PCR, nous avons déterminé les espèces au sein du complexe *Anopheles gambiae*, les infections par *Plasmodium falciparum* au stade sporozoïte et les mécanismes de résistance aux insecticides dans un sous-échantillon d’anophèles vecteurs. *Anopheles gambiae s.l.* est de loin le vecteur majoritaire du paludisme dans la zone de Korhogo. Au total, plus de vecteurs ont été collectés à l’extérieur des habitations qu’à l’intérieur (*p* < 0.001). Des 774 *An. gambiae s.l.* analysés au laboratoire, 89,65 % étaient *An. gambiae s.s.* et 10,35 % *An. coluzzii*. Les fréquences alléliques du gène *kdr* étaient très élevées chez *An. gambiae s.s.* alors que les fréquences alléliques du gène *ace-1* étaient modérées. Une personne non protégée vivant à Korhogo reçoit chaque nuit en moyenne 9,04 piqûres infectantes (pi) en septembre–octobre, 0,63 pi en novembre–décembre, 0,06 pi en février-mars et 0,12 pi en avril–mai. Ces résultats démontrent que l’intensité de la transmission du paludisme est très élevée dans la zone de Korhogo, particulièrement en saison pluvieuse. La lutte contre le paludisme dans les zones de forte endémicité comme Korhogo doit être renforcée par des outils complémentaires afin de réduire le fardeau de la maladie.

## Introduction

Malaria parasites are transmitted to humans by infected female mosquitoes of the genus *Anopheles*, which comprises more than 500 species. About 70 of these species are reported to be vectors of human malaria parasites [38]. The intensity of malaria transmission is exceptionally high in Africa, largely because of the high vectorial capacity of the major vector species, namely *Anopheles gambiae s.s.*, *Anopheles coluzzii, Anopheles arabiensis*, and *Anopheles funestus s.s*. These species display strong anthropophilic host-seeking behavior, and have a long life expectancy, and therefore cause large numbers of secondary malaria cases from one infected individual [41]. The entomological inoculation rate (EIR) that represents the average number of infected bites per person per unit time is generally used to measure the intensity of malaria transmission [39]. The EIR in Africa varies greatly between countries and even within small geographical areas [35]. This heterogeneity needs to be considered when planning and implementing vector control strategies.

Long-lasting insecticidal nets (LLINs) are the main vector control tool used in Côte d’Ivoire to reduce malaria transmission [41]. They are developed to provide physical and chemical barriers against vectors which enter houses to bite humans at night [21]. Consequently, the effectiveness of LLINs may depend on parameters such as the susceptibility of vectors to pyrethroids and their biting behavior. Unfortunately, resistance to the four insecticide classes used for public health (pyrethroids, organochlorines, carbamates, and organophosphates) is common in the main malaria vector species across Côte d’Ivoire [6, 24, 36, 43]. This resistance involves multiple mechanisms such as metabolic and target site resistance. *Kdr* (responsible for dichlorodiphenyltrichloroethane and pyrethroid resistance) and *ace-1* mutations (responsible for organophosphate and carbamate resistance) are widespread, but are found at variable frequencies across the country [6, 24, 36, 43]. However, there is very little recent information on the biting behavior of malaria vector species in many areas of Côte d’Ivoire. Studies in several settings across Africa have shown changes in vector biting behaviors by feeding either mainly outdoors, or in the early morning or in the early evening to avoid contact with insecticide-based tools [14, 29, 32].

Understanding malaria transmission at a local scale by determining the main malaria vector species, their abundance, biting behavior, frequencies of insecticide resistance alleles, and EIRs is a prerequisite for developing and implementing effective control and elimination strategies. The present study was conducted as part of a randomized controlled trial (RCT) in the framework of a project called REACT. The project, which was conducted in 26 villages in northern Côte d’Ivoire (Korhogo area) and 27 villages in southwestern Burkina Faso (Diébougou area), was designed to investigate whether the use of vector control strategies in combination with long-lasting insecticidal mosquito nets in areas with intense pyrethroid-resistance provides additional protection against malaria. In this study, we present the results of one year of entomological investigations in the villages of the Korhogo area in Côte d’Ivoire, with the aim of providing an updated description of malaria transmission and obtaining baseline data for the trial. A preprint of this work was published on March 28, 2019 [42].

## Materials and methods

### Study area

**Figure 1:**
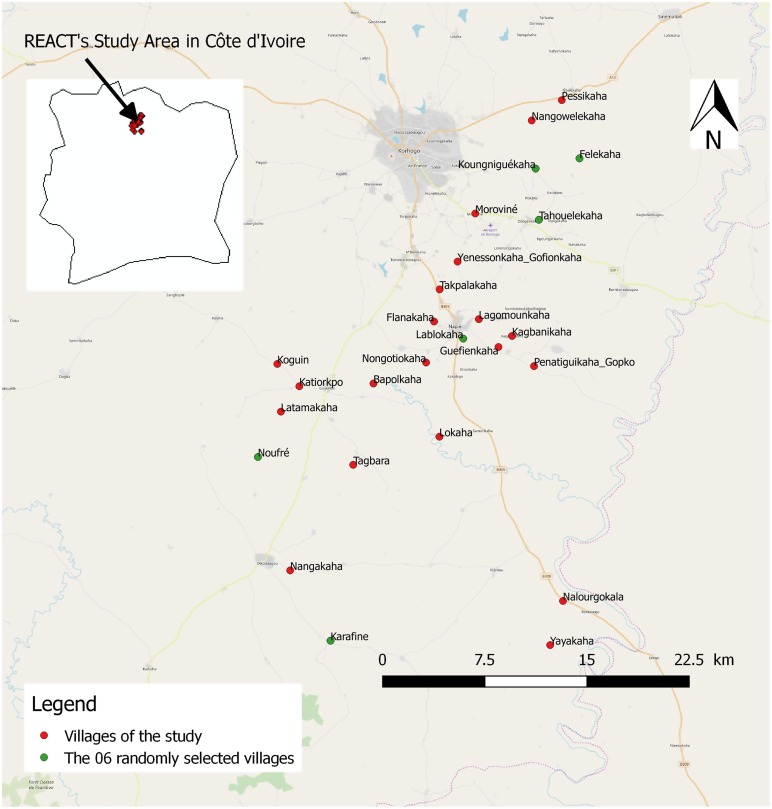
Map of the study area.

The study area included 26 villages of the department of Korhogo (between 9°10′ and 9°40′ N and between 5°20′ and 5°60′ W) located in northern Côte d’Ivoire, West Africa (Fig. 1). The villages were selected based on an average population size of 300 inhabitants, a distance between two villages of at least 2 km, and accessibility during the rainy season. The Korhogo area is characterized by a Sudanese climate with a unimodal rainfall regimen from May to November. The annual rainfall varies from 1200 to 1400 mm, while the mean annual temperature ranges from 21 to 35 °C. The minimum temperatures can drop to 16 °C due to the Harmattan wind during December and January. The natural vegetation is mainly a mixture of savannah and open forest characterized by trees and shrubs that are approximately 8–15 m in height. The Korhogo area is fed by tributaries of the Bandama River such as the Naramou and Solomougou rivers, which dry up in the dry season. Nevertheless, the area has a high density of water dams that allow agriculture to be practiced throughout the year [17]. Therefore, in areas where the soil is highly conducive to agriculture, most of the local inhabitants are farmers and their staple crops include rice, maize, and cotton. Rice is mainly cultivated during the rainy season in flooded soils although it is also occasionally planted in the dry season in irrigated areas in the vicinity of dams. The main malaria vector control intervention in Korhogo is LLINs which are freely distributed by the government every 3–4 years [33]. The last distribution before this study was performed in 2014. According to surveys conducted within the framework of the REACT project, the mean proportion of households with at least one Insecticide Treated Net (ITN) for every two people in the area was 24% in 2016. Indoor residual spraying and larviciding have so far not been implemented in Côte d’Ivoire.

### Mosquito sampling

We performed human landing collections (HLCs) in the 26 villages during four surveys: two in the rainy season (from September 21 to October 10, 2016 and from April 11 to April 29, 2017), and two in the dry season (from November 18 to December 6, 2016 and February 14 to March 04, 2017).

In each village, we collected host-seeking mosquitoes during one night per survey from 17:00 to 09:00 in four randomly selected houses. Each selected house had one collector indoors and one collector outdoors. They were rotated between indoor and outdoor collection sites every hour at each selected house to reduce potential collector bias. Collectors were organized into two teams of eight persons in each village; the first group collected from 17:00 to 01:00 and the second from 01:00 to 09:00. Each night of collection, one technician from the Institut Pierre Richet (IPR) assisted by two local supervisors supervised the mosquito collections in each village to ensure that they were performed properly. The details of the indicators used for quality control are given in Supplementary material.

### *Anopheles* vector processing

After collections, we morphologically identified in the field all the mosquitoes to species or species complex level according to established taxonomic keys [16, 17]. Only *Anopheles* spp*.* mosquitoes were kept and were stored individually in labeled tubes with silica gel at −20 °C for molecular analysis. Due to the very large numbers of vectors collected, we analyzed in the laboratory a subsample of *Anopheles* spp*.* vectors from six villages (Tahouelékaha, Koungniguékaha, Lablokaha, Felékaha, Noufré, and Karafiné) randomly chosen out of the 26 villages included in the study (Fig. 1). From the vectors collected in these six villages, we randomly selected one individual of *Anopheles gambiae s.l.* per hour per collection site during each survey. We analyzed all the *Anopheles nili* complex individuals and all the members of the Funestus Group collected in the six villages. We extracted DNA from the head and thorax of each specimen of the subsample using the Livak method [25]. We identified sibling species of the *Anopheles gambiae* complex by polymerase chain reaction (PCR) [13, 37]. Quantitative PCR (qPCR) was performed to investigate the presence of *kdr* L1014F and *ace-1* G119S mutations in the individuals belonging to the *Anopheles gambiae* complex [2, 12], and to screen all the selected *Anopheles* spp*.* mosquitoes for *Plasmodium falciparum* sporozoite infection [4].

### Ethics approval

Ethical clearance for the study was granted by the national ethics committee (No. 063/MSHP/CNER-kp). We received community agreement before the beginning of the study, and we obtained written informed consent from all the mosquito collectors and supervisors. Yellow fever vaccines were administered to all the field staff. Collectors were treated free of charge when they were diagnosed with malaria during the study period. They were also free to withdraw from the study at any time without any consequences.

### Data analysis

The human biting rate (HBR) for all malaria vector species was calculated as the number of *Anopheles* vectors (*An. gambiae s.l*., *An. funestus* group, *An. nili* complex) collected per person per night. The *P. falciparum* sporozoite rate (SR) in each vector species population was calculated as the number of vectors positive for sporozoites over the number of vectors tested. For each survey, the daily EIR was calculated by multiplying the mean HBR for all malaria vector species by the *P. falciparum* SR in all malaria vector species. Statistical analyses were performed using R software [34].

In order to compare HBRs between surveys and between collection positions (indoors or outdoors), we analyzed counts of *Anopheles* vectors (all species) using a negative binomial mixed effect model (function “glmer” from the package lme4) [5]. The fixed variables were the survey, the collection position (indoors/outdoors), and their interaction. Villages and collection houses were used as random intercepts.

We compared the SR between surveys, species, and collection positions (indoors/outdoors) using a binomial mixed effect model (function “glmer” from the package lme4). The fixed variables were the surveys, the *Anopheles* species, and the collection position (indoors/outdoors). Villages were set as random intercepts.

For both the HBR and SR models, we performed backward stepwise deletion of the fixed terms followed by likelihood ratio tests. We used the Tukey’s *post-hoc* test to compare *Anopheles* counts among surveys according to the final model. Rates ratios, odds ratios and their 95% confidence intervals were computed.

In order to compare night biting activity profiles of *An. gambiae s.l.* among surveys, we compared the distribution of collection times using a Kruskal-Wallis rank sum test, followed by a multiple comparison Dunn’s *post-hoc* test.

We used the G-test [18] implemented in GenePOP 4.7 and run in R [15] to compare gene frequencies at the *kdr* and *ace-1* loci between *An. gambiae s.s*. and *An. coluzzii*, among surveys, and among villages [34]. We tested the distribution of genotypes at the *kdr* and *ace-1* loci for conformity to Hardy-Weinberg equilibrium within both species of the *An. gambiae* complex, in each survey, and in each village, using exact tests as implemented in GenePOP [34].

## Results

We collected a total of 31,324 female mosquitoes belonging to 5 genera (*Anopheles*, *Culex*, *Aedes*, *Mansonia*, and *Uranotaenia*), and 23 species or species complexes/groups in the 26 villages during the four surveys. *Anopheles gambiae s.l.* represented a large proportion of the mosquito species collected, accounting for 94.86%, 59.76%, 74.68%, and 92.12% of the total collected in September–October 2016, November–December 2016, February–March 2017 and April–May 2017, respectively. All the mosquitoes identified as members of the *Funestus* Group and the *An. nili* complex represented less than 2% of the total mosquitoes collected (Table 1). Speciation was successful for 97% of the 764 *An. gambiae s.l.* genotyped to species by PCR. Out of the 356 *An. gambiae s.l.* collected indoors and successfully tested, 314 (88.20%) were *An. gambiae s.s.*, and 42 (11.80%) were *An. coluzzii*. Of the 388 *An. gambiae s.l.* collected outdoors and successfully tested, 353 (90.98%) were *An. gambiae s.s.*, and 35 (9.02%) were *An. coluzzii.* In total, 89.65% of *An. gambiae s.l.* collected and successfully tested were *An. gambiae s.s.*, and 10.35% were *An. coluzzii* (Table 2).

**Table 1:** Diversity and abundance of mosquito species in the 26 villages in the Korhogo area during the four surveys.

Mosquito species	Number of individual mosquitoes collected per survey				
	September–October 2016	November–December 2016	February–March 2017	April–May 2017	Total
*Anopheles gambiae s.l.*	24,831	1344	289	2314	28,778
*Anopheles funestus group*	458	11	0	0	469
*Anopheles nili complex*	75	96	0	6	177
*Anopheles pharoensis*	35	1	1	2	39
*Anopheles ziemanni*	8	2	0	0	10
*Anopheles coustani*	6	1	0	0	7
*Aedes aegypti*	18	14	4	18	54
*Aedes africanus*	4	0	0	0	4
*Aedes furcifer*	3	0	0	0	3
*Aedes hirsutus*	1	0	0	0	1
*Aedes luteocephalus*	37	0	0	0	37
*Aedes vittatus*	2	0	0	0	2
*Culex annuloris*	8	0	0	0	8
*Culex antennatus*	9	6	15	78	108
*Culex cinereus*	119	0	3	0	122
*Culex decens*	4	3	2	0	9
*Culex ingrami*	180	71	40	35	326
*Culex nebulosus*	21	1	1	51	74
*Culex quinquefasciatus*	8	0	0	3	11
*Culex tigripes*	2	0	0	0	2
*Mansonia africana*	221	149	27	5	402
*Mansonia uniformis*	126	549	5	0	680
*Uranotaenia balfouri*	0	1	0	0	1
Total	26,176	2249	387	2512	31,324

**Table 2:** *Anopheles gambiae* complex species in a subset analyzed.

Survey	Species	Number of individuals analyzed	
		Indoor (%)	Outdoor (%)
September–October	*Anopheles gambiae s.s.*	197 (96.10)	211 (99.06)
	*Anopheles coluzzii*	8 (3.90)	2 (0.94)
November–December	*Anopheles gambiae s.s.*	74 (96.10)	86 (97.73)
	*Anopheles coluzzii*	3 (3.90)	2 (2.27)
February–March	*Anopheles gambiae s.s.*	19 (63.33)	24 (61.54)
	*Anopheles coluzzii*	11 (36.67)	15 (38.46)
April–May	*Anopheles gambiae s.s.*	24 (54.55)	32 (66.67)
	*Anopheles coluzzii*	20 (45.45)	16 (33.33)
	*Anopheles gambiae s.s.*	314 (88.20)	353 (90.98)
Total	*Anopheles coluzzii*	42 (11.80)	35 (9.02)
	**Total**	**356**	**388**

The mean HBR for all malaria vector species (*An. gambiae s.l*., *Funestus* Group, *An. nili* complex) in the study area was 35.37 bites per human per night (interquartile range (IQR): 0.00–42.25) during the four surveys. The mean HBR for all malaria vector species was 121.94 (IQR: 68.50–156.50), 6.98 (IQR: 1.00-8.25), 1.39 (IQR: 0.00–1.00), and 11.15 (IQR: 0.00–9.00) bites per human per night in September–October 2016, November–December 2016, February–March 2017 and April–May 2017, respectively. According to the count model, the HBR for all malaria vector species varied significantly between surveys (χ^2^ = 1746.8, df = 3, *p* < 0.001). It was significantly higher in September–October 2016 than in November–December 2016 (OR [95% CI] = 23.82 [18.78, 30.20]), February–March 2017 (OR = 208.60 [155.25, 280.28]), and April–May 2017 (OR = 28.91 [22.07, 37.87]).

### Biting patterns of malaria vector populations

**Figure 2:**
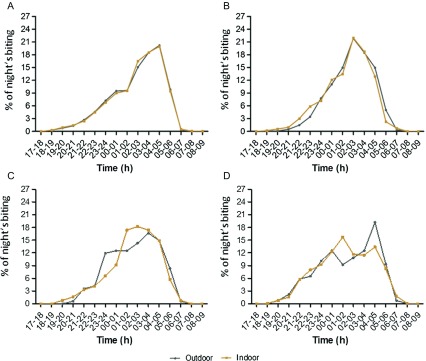
Mean hourly biting activity profiles of *Anopheles gambiae s.l.* mosquitoes in September–October 2016 (A), November–December 2016 (B), February–March 2017 (C), and April–May 2017 (D).

More vectors were collected outdoors than indoors (OR [95% CI] = 1. 26 [1.16–1.37], *p* < 0.001), and the outdoor biting rate did not vary significantly between the surveys (χ^2^ = 3.24, df = 3, *p* = 0.3693). During the four surveys, indoor and outdoor biting activities of *An. gambiae s.l.* occurred from dusk to dawn. Both indoor and outdoor biting activities of *An. gambiae s.l.* peaked between 04:00 and 05:00 in September–October 2016 and between 02:00 and 03:00 in November–December 2016 (Fig. 2A and B). In February–March 2017, the majority of indoor and outdoor bites by *An. gambiae s.l.* occurred between 01:00 and 04:00 and between 03:00 and 04:00, respectively (Fig. 2C). In the April–May 2017 survey, both the indoor and outdoor biting activity curves of *An. gambiae s.l.* showed a bimodal shape. Indoors, there was one peak between 01:00 and 02:00, and a smaller one between 04:00 and 05:00. Outdoors, there was one peak between 00:00 and 01:00, and a higher one between 04:00 and 05:00 (Fig. 2D). The biting activity profiles of *An. gambiae s.l.* differed significantly between surveys (Kruskal–Wallis chi-squared = 217.76, df = 3, *p*-value < 0.001), with activity during the first survey (September–October 2016) peaking later than during the other surveys (Dunn Test *p*-values < 0.001).

### Infection with *P. falciparum* sporozoites and EIRs

*Plasmodium falciparum* sporozoite infection rates per species and surveys are presented in Table 3. The *P. falciparum* sporozoite rate in malaria vector populations was 7.41% (95% IC: 5.47–9.98), 8.98% (95% IC: 5.52–14.29), 4.41% (95% IC: 1.51–12.18), and 1.10% (95% IC: 0.06–5.96) in September–October 2016, November–December 2016, February–March 2017, and April–May 2017, respectively (Table 3). There were no significant differences in the *P. falciparum* sporozoite rates between malaria vector species (χ^2^ = 7.5, df = 3, *p* = 0.057) and surveys (χ^2^ = 4.9, df = 3, *p* = 0.179). *Plasmodium falciparum* sporozoite infection rates in malaria vector populations according to location (indoors and outdoors) are presented in Table 4. The *P. falciparum* sporozoite rates did not differ significantly depending on whether malaria vectors were collected indoors or outdoors (χ^2^ = 0.0796, df = 1, *p* = 0.78).

**Table 3:** *Plasmodium falciparum* sporozoite rates in a subset of *Anopheles* vectors according to taxonomic groups.

Survey	Species, group or complex	No. positive for Pf	No. tested	SR (%) [95% CI]
September–October 2016	*Anopheles gambiae s.s.*	29	408	7.14 [5.02–10.07]
	*Anopheles coluzzii*	1	10	10.00 [0.51–40.42]
	*Funestus* Group	8	47	17.02 [8.89–30.14]
	*Anopheles nili* complex	1	63	1.59 [0.08–8.46]
	**Total**	**39**	**528**	**7.39 [5.45–9.94]**
November–December 2016	*Anopheles gambiae s.s.*	14	160	8.75 [5.28–14.15]
	*Anopheles coluzzii*	1	5	20.00 [1.03–62.45]
	*Funestus* Group	0	1	0.00 [0.00–94.87]
	*Anopheles nili* complex	0	1	0.00 [0.00–94.87]
	**Total**	**15**	**167**	**8.98 [5.52–14.29]**
February–March 2017	*Anopheles gambiae s.s.*	3	43	6.98 [2.40–18.61]
	*Anopheles coluzzii*	0	26	0.00 [0.00–12.87]
	*Funestus* Group	–	0	–
	*Anopheles nili* complex	–	0	–
	**Total**	**3**	**69**	**4.35 [1.49–12.02]**
April–May 2017	*Anopheles gambiae s.s.*	0	55	0.00 [0.00–6.53]
	*Anopheles coluzzii*	1	36	2.78 [0.14–14.17]
	*Funestus* Group	–	0	–
	*Anopheles nili* complex	–	0	–
	**Total**	**1**	**91**	**1.10 [0.06–5.96]**

No.: number of mosquitoes; Pf: *Plasmodium falciparum*; SR: Sporozoite rate; [95% CI]: 95% Wilson’s confidence interval.

**Table 4:** *Plasmodium falciparum* sporozoite rates in a subset of *Anopheles* vectors according to locations (indoors and outdoors).

	Location	No. positive	No. tested	SR (%) [95% CI]
September–October	Indoor	17	238	7.14 [4.51–11.14]
	Outdoor	22	290	7.59 [5.06–11.22]
	**Total**	**39**	**528**	**7.39 [5.45**–**9.94]**
November–December	Indoor	7	77	9.09 [4.47–17.60]
	Outdoor	8	90	8.89 [4.57–16.57]
	**Total**	**15**	**167**	**8.98 [5.52**–**14.29]**
February–March	Indoor	2	30	6.67 [1.85–21.32]
	Outdoor	1	39	2.56 [0.13–13.18]
	**Total**	**3**	**69**	**4.35 [1.49**–**12.02]**
April–May	Indoor	0	44	0.00 [0.00–8.03]
	Outdoor	1	47	2.13 [0.11–11.11]
	**Total**	**1**	**91**	**1.10 [0.06**–**5.96]**

No.: number of mosquitoes; SR: Sporozoite rate; [95% CI]: 95% Wilson’s confidence interval.

Overall, an unprotected individual living in the Korhogo area was estimated to receive an average of 9.04, 0.63, 0.06 and 0.12 infected bites per night in September–October 2016, November–December 2016, February–March 2017, and April–May 2017, respectively. In September–October 2016, an estimated 0.69 infected bites per night (i.e., 20.7 per month) occurred between 17:00 and 22:00. The average EIR recorded in this study was 2.46 infected bites per person per night.

### Frequencies of the *kdr* and *ace-1* alleles

The frequency of the *kdr* allele in the *An. gambiae s.s.* population (frequency range: 90.70–100%) was very high throughout the study periods (Table 5). It was significantly higher in September–October 2016 than in February–March 2017 (*p* < 0.001) and April–May (*p* < 0.001). The frequency of the *kdr* allele in the *An. gambiae s.s.* population in September–October 2016 did not differ from that of November–December 2016 (*p* = 1.00). The frequency of the *kdr* allele in the *An. coluzzii* population ranged from 55.6% to 100% during the four surveys (Table 5). It was significantly higher in September–October 2016 than in February–March 2017 (*p* < 0.001) and April–May 2017 (*p* < 0.001). The frequency of the *kdr* allele in the *An. coluzzii* population did not vary significantly between September–October 2016 and November–December 2016 (*p* = 1.00). The *kdr* allele was more frequent in the *An. gambiae s.s.* than in the *An. coluzzii* population (*p* < 0.001). We found significant variations in the frequency of the *kdr* allele in the *An. gambiae s.s.* (*p* < 1 × 10^−4^) and *An. coluzzii* (*p* = 0.020) populations among the six villages. For each survey, genotype frequencies of *kdr* in the *An. gambiae s.s* and *An. coluzzii* populations from each of the six villages were not different from the Hardy–Weinberg expectations (*p* > 0.05).

**Table 5:** *Kdr* mutation frequencies in a subset of *Anopheles gambiae s.l.*

		RR	RS	SS	Total	Allelic frequency (%) [95% CI]
September–October	*Anopheles gambiae s.s.*	402	1	0	403	99.88 [99.30–99.99]
	*Anopheles coluzzii*	9	1	0	10	95 [76.39–99.74]
November–December	*Anopheles gambiae s.s.*	160	0	0	160	100 [98.81–100]
	*Anopheles coluzzii*	5	0	0	5	100 [72.25–100]
February–March	*Anopheles gambiae s.s.*	37	4	2	43	90.70 [82.70–95.21]
	*Anopheles coluzzii*	11	11	4	26	63.46 [49.87–75.20]
April–May	*Anopheles gambiae s.s.*	52	2	2	56	94.64 [88.80–97.52]
	*Anopheles coluzzii*	12	16	8	36	55.56 [44.09–66.46]

RR: homozygous resistant; RS: heterozygote; SS: homozygous susceptible; [95% CI]: 95% Wilson’s confidence interval.

The *ace-1* frequency in the *An. gambiae s.s.* population ranged from 15.18 to 30.05% throughout the study periods (Table 6). The frequency of the *ace-1* allele in the *An. gambiae s.s.* population was significantly higher in September–October 2016 than in November–December 2016 (*p* = 0.021), February–March 2017 (*p* = 0.026) and April–May 2017 (*p* < 0.001). The frequency of the *ace-1* allele in the *An. coluzzii* population (frequency range: 15.18–30.05%) did not vary significantly among the four surveys (*p* = 0.278). The *ace-1* allele was significantly more frequent in the *An. gambiae s.s.* than in *An. coluzzii* population (*p* < 1 × 10^−4^). The frequency of the *ace-1* allele in the *An. gambiae s.s.* population varied significantly among the six villages (*p* < 1 × 10^−4^). The frequency of the *ace-1* allele in the *An. coluzzii* population did not vary significantly among the six villages (*p* = 0.211). For each survey, genotype frequencies of *ace-1* in the *An. gambiae* and *An. coluzzii* populations from each of the six villages did not differ from the Hardy–Weinberg proportions (*p* > 0.05).

**Table 6:** *Ace1* mutation frequencies in a subset of *Anopheles gambiae s.l.*

		RR	RS	SS	Total	Allelic frequency (%) [95% CI]
September–October	*Anopheles gambiae s.s.*	41	156	199	396	30.05 [26.96–33.33]
	*Anopheles coluzzii*	0	1	9	10	5 [0.26–23.61]
November–December	*Anopheles gambiae s.s.*	15	43	101	159	22.96 [18.67–27.88]
	*Anopheles coluzzii*	0	0	5	5	0 [0–27.75]
February–March	*Anopheles gambiae s.s.*	2	12	29	43	18.60 [11.79–28.10]
	*Anopheles coluzzii*	0	0	26	26	0 [0–6.88]
April–May	*Anopheles gambiae s.s.*	3	11	42	56	15.18 [9.70–22.97]
	*Anopheles coluzzii*	0	1	35	36	1.39 [0.07–7.46]

RR: homozygous resistant; RS: heterozygote; SS: homozygous susceptible; [95% CI]: 95% Wilson’s confidence interval.

## Discussion

*Anopheles gambiae s.l.* is by far the main malaria vector in the Korhogo area, irrespective of the season, but its abundance varied significantly according to the season. The risk of being bitten by malaria vector mosquitoes was found to be up to 200-fold higher in September–October 2016 (rainy season) than in February–March 2017 (dry season). These results are consistent with the bionomics of *An. gambiae s.s*. Indeed, the breeding habitats of this species are known to increase in number and productivity during the rainy season but almost disappear during the dry season [30]. In September–October 2016 (rainy season), an unprotected individual in the Korhogo area received an estimated nine infected bites per night (i.e., 270 per month). Korhogo is a rural area where several crops including rice are cultivated. Rice paddies are strongly associated with very high densities of malaria vectors [20]. In this study, we found a mean annual EIR of 897.9 infected bites per person per year. Research conducted so far in Africa has reported mean annual EIRs ranging from <1 to >1000 infected bites per person per year [3]. Therefore, Korhogo ranks at the top level among malaria endemic areas in terms of the intensity of transmission.

Our results revealed that malaria vector populations were significantly exophagic in both the rainy and dry seasons, with no significant difference in outdoor biting rates between seasons. Furthermore, we found that indoor and outdoor biting activities of *An. gambiae s.l.* peaked later (4–5 am) in September–October 2016 than in the other three surveys. The influence of genetic factors in the biting behaviors of malaria vectors remains poorly understood [26]. However, some studies have shown that *Anopheles* vectors can adjust their biting behavior in response to a small change in environmental parameters such as temperature [9, 31]. During the four surveys, we recorded environmental data (temperature, humidity, atmospheric pressure, and light intensity) at all the sites during collection. These data would help establish a possible link between environmental variables and the biting behaviors of vectors. It is more and more obvious that LLINs alone are unable to eliminate malaria in high malaria burden countries [19]. In this study, we found that an estimated 0.69 infected bites (i.e., 20.7 per month) occurred daily in September–October 2016 between 17:00 and 22:00 when human populations are potentially unprotected by ITNs. Nonetheless, our results need to be adjusted to the behaviors of the local human populations in terms of sleeping hours, outdoor activities and ITN use in order to more accurately measure human exposure to *Plasmodium* sp*.-*infected mosquito bites in the Korhogo area [28]. We will address this research question in another publication using additional data collected in the framework of the REACT project.

Consistent with recent studies carried out in the Korhogo area [6, 24], we found very high frequencies of the *kdr* mutation in the *An. gambiae* complex population during the four surveys. The *kdr* mutation has almost reached fixation in *An. gambiae s.s.* population across Côte d’Ivoire [6]. We identified the *ace-1* allele in the *An. gambiae* complex populations at frequencies ranging from 0 to 30.05% during the four surveys. A recent study has shown that *An. gambiae s.l.* mosquitoes from Korhogo are highly resistant to pyrethroids, organochlorides, and carbamates, but susceptible to organophosphates [6]. Accordingly, the use of organophosphates may represent a valuable strategy for the control of this high insecticide resistant vector population. However, this approach would select genotypes bearing *ace-1* mutations which display resistance to organophosphates although the *ace-1* mutation confers a lower resistance level to organophosphates than carbamates [11]. In our study, we found spatial and temporal variations of the *kdr* and *ace-1* frequencies in the *An. gambiae* complex population. The frequencies of insecticide resistance alleles in malaria vector populations are known to be influenced by the selective pressures from insecticides used in agriculture and public health, as well as by the fitness costs associated with resistance alleles in the absence of insecticide [1, 10, 23, 27, 40]. Both the *kdr* and *ace-1* alleles were significantly more frequent in the *An. gambiae s.s.* than in the *An. coluzzii* population. These differences in *kdr* and *ace-1* frequencies between *An. gambiae s.s.* and *An. coluzzii* have been observed in many areas of Africa [7, 8, 12].

This study demonstrated that malaria vectors were more likely to feed outdoors than indoors, and the frequencies of the *kdr* allele were very high in *An. gambiae s.l.* populations. Moreover, we found that the risk of malaria transmission was significant when people are potentially active. Therefore, malaria transmission would still continue in the Korhogo area even with full coverage of LLINs. A large-scale multi-country study published recently showed conclusively that LLINs are still protective against malaria despite widespread resistance to pyrethroids [22]. However, malaria control in high endemic areas such as Korhogo needs to be strengthened with complementary tools to alleviate the burden of the disease. Therefore, efforts need to be dedicated to evaluating the effectiveness of promising tools to be used in combination with LLINs in high endemic areas. The REACT project will provide data on the impact of four potential strategies on clinical malaria and transmission in two high endemic areas of Burkina-Faso and Côte d’Ivoire. Two of these strategies, i.e., larviciding with *Bti* and use of ivermectin in cattle and other small ruminants, are in late stages of development, while the two others, i.e., intensive communication for human behavioral changes and indoor residual spraying of insecticide, are already available in the vector control arsenal against malaria.

## Conclusions

Malaria transmission in the Korhogo area was mainly due to *An. gambiae s.s.* and *An. Coluzzii*, while *An. funestus* group species and *An. nili* complex species played minor roles. The frequencies of the *kdr* mutation in the *An. gambiae* complex population were very high, whereas *ace-1* frequencies were moderate. Malaria vectors in Korhogo were more exophagic. The intensity of malaria transmission is extremely high in the Korhogo area, especially during the rainy season. Malaria control in such highly endemic areas needs to be strengthened with complementary tools in order to reduce the burden of the disease.
