# Supplementary material for: Anopheles bionomics, insecticide resistance mechanisms, and malaria transmission in the Korhogo area, northern Côte d’Ivoire: a pre-intervention study
Source: Parasite. 2019 Jul 12;26:40. doi: 10.1051/parasite/2019040 (PMC6625791; doi:10.1051/parasite/2019040)
Supplement: Supplementary file 1 [file parasite-26-40-olm.pdf]

Quality control of mosquito collections

Date: **Village:** **House number:** **0: satisfactory**

SUPERVISOR: **1: unsatisfactory**

	INDICATORS	17-18H	18-19H	19-20H	20-21H	21-22H	22-23H	23-24H	00-01H	01-02H	02-03H	03-04H	04-05H	05-06H	06-07H	07-08H	08-09H
Indoors	Clothing																
	Presence at the site																
	Awake state																
	Position																
	Rotation between collectors																
	Presence of measuring devices																
	Position of measuring devices																
Outdoors	Clothing																
	Presence at the site																
	Awake state																
	Position																
	Rotation between collectors																
	Presence of measuring devices																
	Position of measuring devices																
